# Zero-Gap Electrochemical CO_2_ Reduction Cells: Challenges and Operational Strategies for Prevention of Salt Precipitation

**DOI:** 10.1021/acsenergylett.2c01885

**Published:** 2022-12-05

**Authors:** Mark Sassenburg, Maria Kelly, Siddhartha Subramanian, Wilson A. Smith, Thomas Burdyny

**Affiliations:** † Materials for Energy Conversion and Storage (MECS), Department of Chemical Engineering, Delft University of Technology, 2629 ZHDelft, The Netherlands; ‡ Department of Chemical and Biological Engineering and Renewable and Sustainable Energy Institute (RASEI), University of Colorado Boulder, Boulder, Colorado80303, United States; § National Renewable Energy Laboratory, Golden, Colorado80401, United States

## Abstract

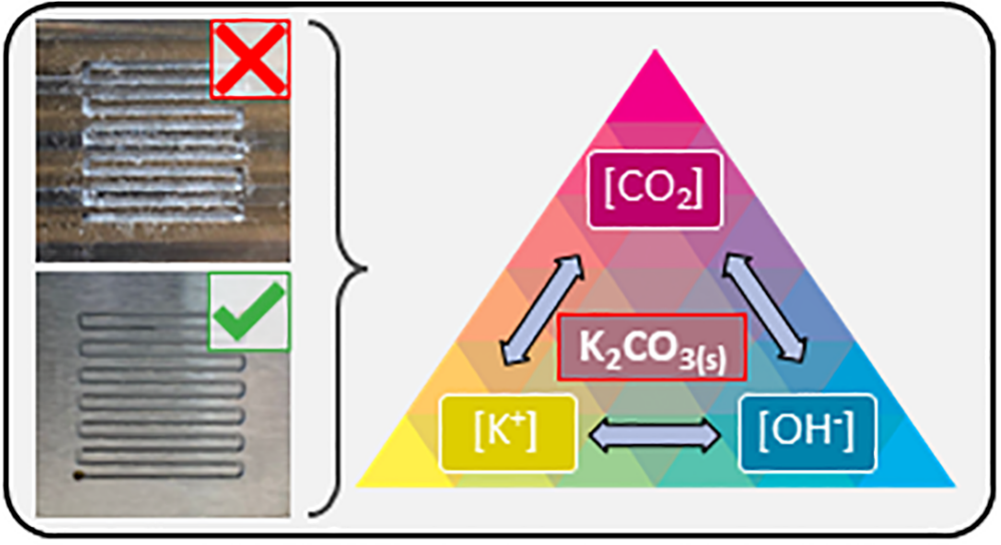

Salt precipitation is a problem in electrochemical CO_2_ reduction electrolyzers that limits their long-term durability and industrial applicability by reducing the active area, causing flooding and hindering gas transport. Salt crystals form when hydroxide generation from electrochemical reactions interacts homogeneously with CO_2_ to generate substantial quantities of carbonate. In the presence of sufficient electrolyte cations, the solubility limits of these species are reached, resulting in “salting out” conditions in cathode compartments. Detrimental salt precipitation is regularly observed in zero-gap membrane electrode assemblies, especially when operated at high current densities. This Perspective briefly discusses the mechanisms for salt formation, and recently reported strategies for preventing or reversing salt formation in zero-gap CO_2_ reduction membrane electrode assemblies. We link these approaches to the solubility limit of potassium carbonate within the electrolyzer and describe how each strategy separately manipulates water, potassium, and carbonate concentrations to prevent (or mitigate) salt formation.

The electrochemical CO_2_ reduction reaction (CO_2_RR) provides a pathway toward a more CO_2_ neutral society. Although still in its infancy, the potential for this technology to develop further has led to improvements in the product selectivity, activity, and stability of CO_2_RR electrolyzers. Much has been adopted from the already matured electrochemical hydrogen evolution reaction (HER) field, where performance metrics such as >1 A cm^–2^ conversion and >10,000 h lifetime are easily surpassed.^1^ By adopting technical features like the gas diffusion electrode (GDE)^2−6^ and membrane electrode assembly (MEA)^7^ cell architecture, the field of CO_2_ reduction has achieved industrially relevant current densities (>200 mA cm^–2^) while retaining selective conversion. These improvements were in part realized by utilizing highly alkaline electrolytes, such as KOH, to limit the competing HER^4,8−10^ and humidifying the CO_2_ gas stream to manage water availability to the cathode and membrane.^11,12^ However, since being incorporated into more industrial reactors, additional challenges have been found which impact the long-term stability and economic feasibility of CO_2_RR. In particular, the precipitation of salts within the reactor leads to operational failures which diminish the potential impact of this technology. In higher energy efficiency MEA architectures where a liquid catholyte is removed, salt precipitation is common and highly disruptive to steady performance.

An exchange MEA is the most common MEA architecture used in CO_2_RR electrolyzers. The cathode side uses a porous GDE and is fed with a gaseous stream of CO_2_ that can be dry or humidified. The anode of the exchange MEA contacts a liquid anolyte that provides reactants for the anode reaction (typically oxygen evolution) and serves as a water source for the membrane.^12^ MEAs with a gaseous anode feed (also known as full MEAs) have been demonstrated for CO_2_RR,^13−16^ but reports on these systems are limited and fall outside the primary scope of this Perspective.

The use of GDEs in MEAs is the feature which enables elevated current densities by reducing the liquid diffusion length of CO_2_ from the gas phase to the catalyst surface. However, the production of hydroxide as a byproduct of CO_2_RR during water-splitting, and the use the KOH as an anolyte, result in a highly alkaline local environment^17−19^ in the cathode compartment of the electrolyzer. The excess CO_2_ which is enabled by the gas-diffusion layer then simultaneously provides a route toward salt formation through the production of (bi)carbonates (Figure 1).

**Figure 1:**
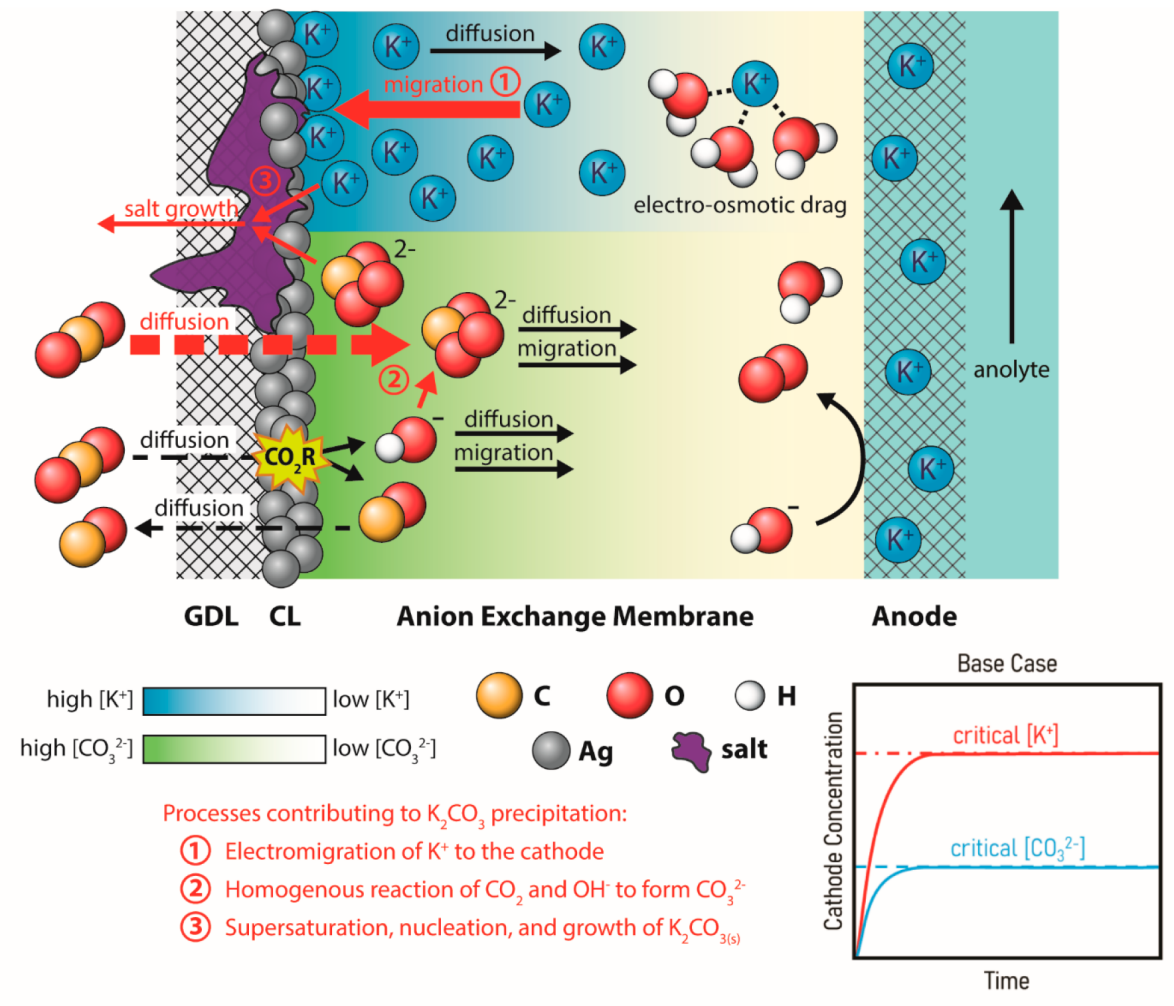
Schematic representation of the cascade of reactions and ion transport in an exchange MEA leading to salt formation on the cathode composed of a catalyst layer (CL) and gas-diffusion layer (GDL). The inserted graph shows the change in ion concentrations occurring near the cathode. After both CO_3_^2–^ and K^+^ concentrations reach critical levels, the precipitation of K_2_CO_3_ starts to occur.

In MEAs, these carbonate salts can form in the cathode flow field, on the gas side of the cathode, within the GDE, and on the membrane side of the electrode in systems using both alkaline and near-neutral anolytes.^7,12,20,21^ The deposits block the initially porous GDE and cause the pressure within the cathode chamber to increase as gas flow is progressively restricted by the salts.^20,22^ The presence and formation of salt also restricts access of CO_2_ to the catalyst, leading to increased hydrogen Faradaic efficiencies. Although salt precipitation has been observed in other alkaline electrochemical systems,^23,24^ its prevalence in CO_2_ reduction electrolyzers comes from the interplay of 3 components essential to CO_2_RR: the reactant CO_2_ gas, the proton-source (H_2_O or HCO_3_^–^),^25−27^ and a cation that assists in catalysis.^28−30^ Several citations used in the work presented here make use of 3 compartment flowcells^22,31,32,41,56^ and even fully aqueous setups,^25−30,47^ in which mass transport can be quantitatively different. Nevertheless, the underlying principles of local alkalinity, water, and ion transport can generally be translated to MEA systems.

Several operational approaches have been deployed in literature to maintain long-term CO_2_ electrolysis without salt formation. In essence, however, each of these strategies work toward a similar goal and separately prevent salt formation by lowering either [K^+^], [CO_3_^2–^], or [K_2_CO_3_] in the cathode compartment. Some are “active” approaches that require a periodic change in the operational state of the electrolyzer. Others are “passive” approaches that are in effect at all times. Here, we group the strategies presented in literature into four general categories. (1) Passive Anolyte Approach: the anolyte concentration is decreased, or the cation identity is changed, to keep the accumulation of cations at the cathode surface below the critical salting out concentration. (2) Active Dissolution Approach: the cathode is periodically pulsed with water or an equivalent solvent to dissolve accumulated salts and increase water availability. Alternatively, while feeding a deionized water anolyte, the cathode is periodically flushed with an “activation” solution to provide cations near the cathode surface. (3) Active Pulse Approach: the MEA is operated in a pulsed electrolysis mode where periodically switching to a low applied potential allows accumulated cations and carbonate ions to diffuse away from the cathode, thereby keeping their concentration below critical levels. (4) Passive Membrane Approach: the MEA membrane and its components are chosen to reduce ion migration to and accumulation at the cathode.

This Perspective reflects on these operational strategies for avoiding or reversing salt formation in CO_2_ electrolyzers. We discuss each of these approaches in-depth next to the phenomena causing salt formation to highlight that all strategies work toward the same goal of avoiding the solubility limits of carbonate salts, each by targeting either the cation, anion, or water concentrations.

First, to explain how salt formation takes place, we look at the conversion of CO_2_-to-CO on a Ag catalyst in an alkaline environment. During electrolysis, some of the CO_2_ fed into the system is converted to CO as described by the cathodic half reaction:
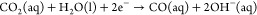
1

For each converted CO_2_ molecule, two hydroxide ions are produced when in a neutral or alkaline pH environment. In addition to making the environment more alkaline, OH^–^ also participates in the unwanted homogeneous conversion of CO_2_ to bicarbonate and carbonate (depending on the exact pH):
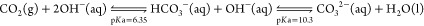
2

Since CO_2_ gas is abundantly present and hydroxides are continuously produced, the effectively utilized amount of CO_2_ gas for CO_2_RR can drop down to ∼30% due to dissolution, while up to ∼70% of CO_2_ is converted into carbonates that can fuel salt formation.^31,32^ Multiphysics models developed by Weng et al. and Kas et al. have also determined the maximum CO_2_ utilization efficiency to be ∼50% for an exchange MEA system and a GDE with a flowing catholyte, respectively.^33,34^ While this is a significant problem on its own in terms of CO_2_ utilization efficiency, another issue is the accumulation of carbonate at the cathode due to reaction 2.

The third reaction to consider is the combination of accumulating carbonate ions near the gas–liquid interface and the cations (i.e., K^+^) that are used to improve ionic conductivity and stabilize CO_2_ reduction intermediates. Since the cathode is negatively charged during electrolysis and hydroxide ions are being produced, migration of cations from the anolyte past the membrane leads to a gradually increasing concentration near the cathode to maintain charge neutrality within the system. Ultimately the high concentrations of cations and carbonates exceed the solubility limit (1096 g/L or 7.93 M K_2_CO_3_ at 20 °C in pure water)^35^ and lead to the formation of salts:
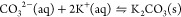
3

It is most accurate to use the solubility product constant (*K*_sp_) to define the conditions for K_2_CO_3_ precipitation. However, K_2_CO_3_ is highly soluble, and at saturation the solution would deviate from ideal solution behavior. For simplicity, the remainder of this review will use the solubility of K_2_CO_3_ in units of molarity to describe the conditions for precipitation with the disclaimer that greater concentrations of potassium and carbonate could lead to earlier than described salt formation. For this reason, operational strategies should aim to keep both K^+^ < 15.86 M and CO_3_^2–^ < 7.93 M to avoid the solubility product from exceeding the solubility limit.

In addition to K_2_CO_3_, KHCO_3_ and K_4_H_2_(CO_3_)_3_·1.5H_2_O^20^ have also been detected by ex situ XRD in MEA cathodes. KHCO_3_ and K_4_H_2_(CO_3_)_3_·1.5H_2_O can form by CO_2_ sorption of solid K_2_CO_3_, so it is proposed that K_2_CO_3_ initially precipitates then reacts with excess CO_2_ in the gas stream to form other carbonate salts.^20,36^

Many studies have examined the effect of different salt cations on the performance of CO_2_RR systems, but here we focus on the implications of K^+^ as it is the most studied salt cation. These conclusions can be generalized to other cations, albeit with different solubility limits potentially changing the primary location of salt formation in the cathode compartment.

While the chemical reactions in eqs 1–3 describe how ions are formed and precipitate into salts, the Nernst–Planck equation then describes the transport and accumulation of ions across the electrochemical system:
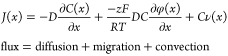
4where *J*(*x*) is the flux of an ionic species, *D* is its diffusivity constant, d*C*/d*x* is the concentration gradient, *z* is its electronic charge, *F* is Faraday’s constant, *R* is the ideal gas constant, *T* is the temperature, dϕ/dx is the electrical potential gradient, and *v*(*x*) is the fluid velocity. Near the electrode surface where the fluid velocity ν is negligible (*Cν*(*0*) = 0), this equation states that in a steady state system where there is no net flux of ionic species (*J*(*x*) = 0), the electromigration of potassium ions toward the negative cathode has to equalize with the diffusion of high concentrations back to the (relatively) low concentration bulk.

Within a zero-gap system the concentrations of ionic species are then determined by the applied reaction rate, the anolyte concentration, and the diffusion, migration, and convection driven ionic transport through the cathode region, membrane, and anode region. While carbonate forms easily as gaseous CO_2_ reacts with the OH^–^ product (eq 2), a zero-gap system typically has limited potassium ions initially at the cathode. Moreover, the majority of reported zero-gap systems utilize anion exchange membranes, which should be repellant to cations.^37^ Driven by the high concentration of negative charges at the cathode, counterion transport of potassium across the anion exchange membrane is facilitated through electro-osmotic drag as depicted in Figure 1. In conjunction with water transport, partially neutralized potassium ions are able to cross the membrane and accumulate at the cathode.

In order to avoid potassium carbonate precipitation in a strongly alkaline system, the concentrations of both CO_3_^2–^ and K^+^ must be kept below 7.93 and 15.86 M, respectively. Although these concentrations are much higher than the ∼1 M K^+^ of typical CO_2_RR electrolytes, the substantial production of hydroxide and carbonate at elevated current densities creates such an environment, as was computationally hypothesized by several catalyst layer concentration models.^17,38,39^

The experiences of rapid salt formation at industrially relevant current densities (e.g., 50 min for a 2 M KOH anolyte operating at 100 mA cm^–2^)^40^ indicate that the migration term of cations toward the cathode is larger than the diffusion term in eq 4. Once salting out conditions are met, nucleation occurs and rapid growth of crystals is observed into the cathode pores and flow field until salts block gas flow altogether.

To achieve an operational lifetime in the range of hydrogen electrolyzers (>10,000 h), methods for the prevention (or reversal) of salt formation in CO_2_RR MEA systems need to be developed and improved. However, MEA designs to prevent carbonate precipitation faces several challenges with various trade-offs for performance and durability. Any change made to suppress salt formation often contributes to other negative effects such as electrolyte flooding,^41^ loss of CO_2_RR selectivity over HER,^42^ increase in cell voltage,^43^ or increased down time of the reactor for cleaning or pulsed electrolysis modes.^44^ Thus, implementation of engineering and design methods for precipitation prevention results in a complex optimization problem of many MEA operational factors.

In the past decade of CO_2_RR research, salt precipitation in CO_2_ electrolyzers with GDEs has not been studied extensively despite being a commonly observed phenomenon. Only a few papers have mentioned salt formation and its importance in operations, while fewer provide empirical engineering solutions to obtain longer stability. By analyzing the research that has sought to overcome salt precipitation we were able to identify 4 main categories of engineering solutions. These approaches include (i) passively modifying the anolyte concentration and composition, (ii) actively dissolving salts at the cathode, (iii) actively pulsing the electrolyzer, and (iv) passively modifying the MEA. Collectively, these strategies tackle the same issue of preventing potassium and carbonate from simultaneously reaching their critical concentrations.

## 1 Passive Anolyte Approach: Cation Concentration and Identity

**Figure 2:**
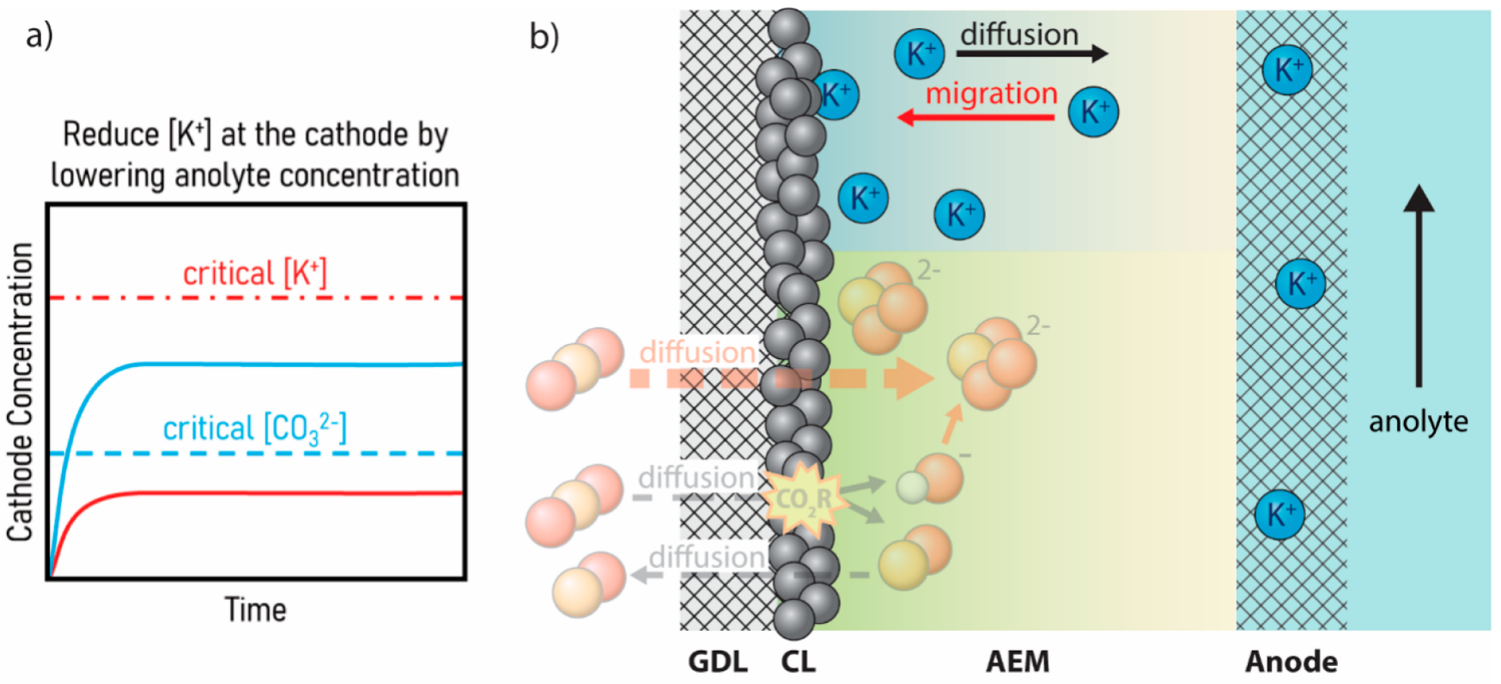
(a) Plot of cathode concentration versus time showing the general trends of K^+^ and CO_3_^2–^ concentrations at the cathode when the anolyte concentration is reduced. (b) Schematic depiction of a lower concentration of K^+^ in the anolyte solution resulting in reduced electromigration. This enables the balancing between migration and diffusion of K^+^, keeping the total concentration below the solubility limit of K_2_CO_3_.

The first option presented to reduce salt formation is to decrease the concentration of cations in the electrolyte, or eliminate them entirely from the system (illustrated in Figure 2). From a mass transport perspective, a lower bulk concentration of K^+^ in the anolyte reduces the transport effects of ion migration from the anode to the cathode. Migration is then balanced by diffusion of cations from the cathode to the anode. Combined, the accumulation of potassium at the cathode is maintained below the solubility limit of K_2_CO_3_, thereby preventing salt precipitation (Figure 2a).

Liu et al. showed that reducing the anolyte concentration to 10 mM KHCO_3_ instead of the typical 1 M concentration allowed stable operation for 3800 h (200 mA cm^–2^, 3 V_cell_).^43^ In this situation, the diffusion and migration terms equalize and keep the potassium concentration below the critical salting out condition. However, the use of a lower anolyte concentration also increased the overall cell resistance, leading to higher cell potentials. Similarly, Endrődi et al. observed that decreasing the electrolyte concentration prolongs electrolyzer operation at the expense of current density. When operating an MEA at 3.1 V_cell_, the current density with a 0.1 M KOH anolyte was 300 mA cm^–2^ but dropped to 100 mA cm^–2^ in a deionized water anolyte.^20^ The drop in current density when using a pure water feed can again be attributed to its low conductivity: electrochemical impedance spectra of both cells indicated a 3 to 4 times larger charge transfer resistance in the MEA fed with pure water compared to 0.1 M KOH.

However, the performance of CO_2_RR MEAs using a pure water anolyte has been improved using novel membranes and ionomers. For example, Yin et al. used a quaternary ammonia poly(*N*-methylpiperidine-*co*-*p*-terphenyl) polymer as both anion exchange membrane and cathode ionomer in an MEA operating with pure water anolyte. The system achieved 100 mA cm^–2^ at 2.25 V for over 100 h with CO FE consistently greater than 90%.^45^ The same system reached 500 mA cm^–2^ and ∼90% FE at 3 V and 60 °C, although long-term durability at this current density was not reported. By avoiding the use of an alkaline electrolyte and consequently the introduction of metal cations, the authors were able to prevent salt precipitation entirely. Notably, it is generally agreed upon that small amounts of alkali metal cations are needed to increase the system conductivity and stabilize the CO_2_RR intermediates,^29^ so the mechanisms for CO_2_RR in systems with deionized water anolytes should be further investigated. O’Brien et al. suggests such systems without a mobile cation can still achieve high CO_2_RR selectivity if the fixed positive charges in the anion exchange membrane are able to stabilize the CO_2_ reduction intermediates instead.^46^

These examples demonstrate the trade-off between salt precipitation and cell voltage when lowering the anolyte concentration. Thus, for the issue of salt prevention, the question is whether it is economically beneficial to prevent salt precipitation by using dilute electrolytes that will increase the overall cell potential. As more data on long-term testing of CO_2_RR electrolyzers becomes available, technoeconomic analyses should consider the trade-off between cell potential and cell lifetime which is influenced by salt precipitation. As an alternative approach to limit potassium crossover from the anode, the properties of anion exchange membranes themselves could also play an important role. By varying the thickness, water permeability, hydration, and ionic resistances,^47,48^ modifications of the membrane may limit potassium crossover without reduction of anolyte concentration.

Salt precipitation may also be controlled by altering the cation identity of the anolyte. Cofell et al. observed that switching the electrolyte from KOH to CsOH in a flow cell resulted in smaller, well-dispersed bicarbonate crystal deposits and a slowing of the performance degradation caused by the precipitation of carbonate salts.^4^ By contrast, the bicarbonate deposits formed from the KOH electrolyte covered much larger areas of the cathode and formed fractal-like patterns. Chiacchiarelli et al. also noted the effect of cation identity on slowing the formation of deposits on an electrode.^49^ In their work, a rotating Sn electrode was submerged in a 0.1 M KHCO_3_ electrolyte purged with N_2_. Subsequent electrolysis resulted in several degradation modes, including alkali deposits from the electrolyte. The amount of the deposits decreased based on the cation identity in the order Na^+^ > K^+^ > Cs^+^. This trend could be explained by the solubility change with cation identity (Table 1). For carbonates, the solubility (in units of molarity) increases in the order Na^+^ < K^+^ ≈ Cs^+^, and for bicarbonates, the trend is Na^+^ < K^+^ < Cs^+^.^35^ Additionally, differences in ionic radius, ion hydration, and ion diffusivity have all been suggested to affect the rate of cation and water transport to the cathode surface and the energies required to nucleate and grow a carbonate salt.^4,49^ These effects of cation identity on salt precipitate morphology merit further investigation and have yet to be shown in an MEA architecture.

**Table 1:** Solubility of (Bi)carbonate Species for Na^+^, K^+^, and Cs^+^ Cations

salt	solubility (M at 20 °C)
NaHCO_3_	1.14
KHCO_3_	2.24
CsHCO_3_	3.49
Na_2_CO_3_	2.06
K_2_CO_3_	7.93
Cs_2_CO_3_	8.01

## 2 Active Dissolution Approach: Adding Solvents to the Cathode

**Figure 3:**
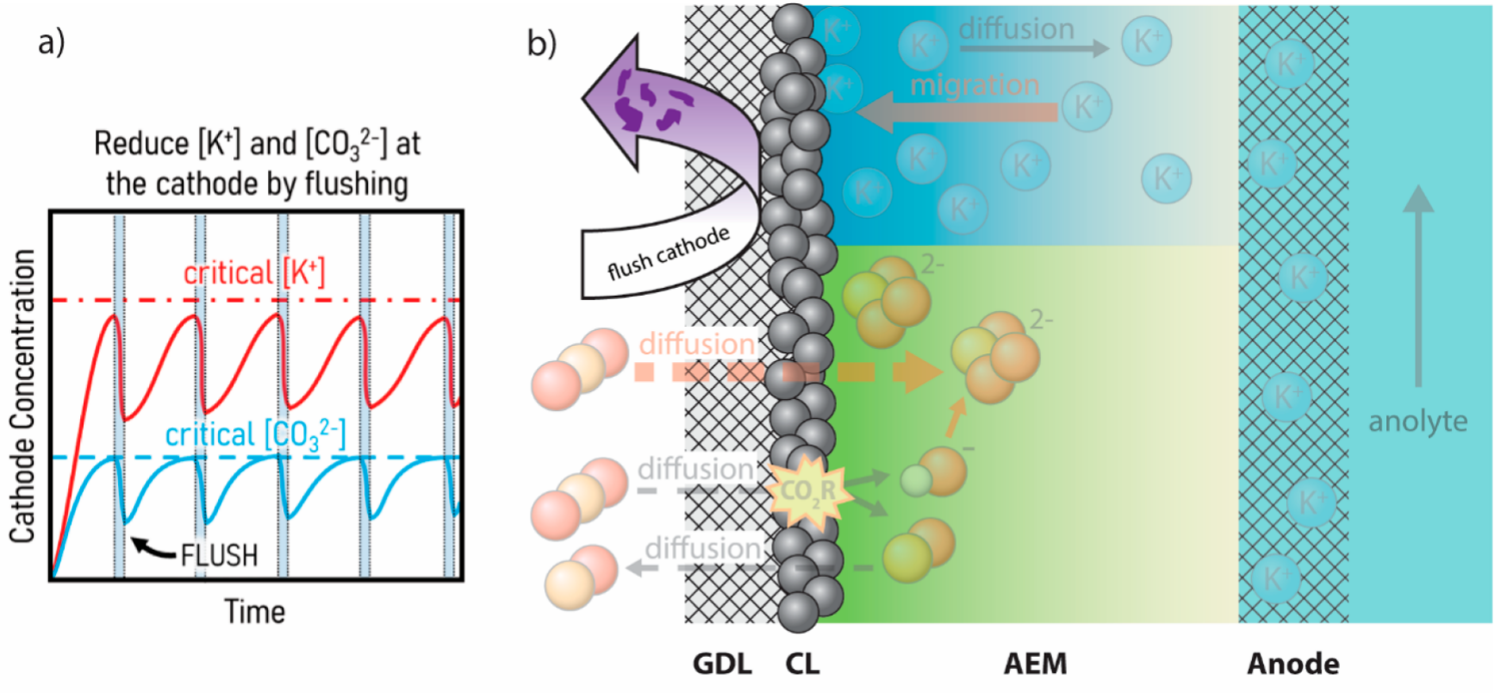
(a) Plot of cathode concentration versus time showing the general trends of K^+^ and CO_3_^2–^ concentrations at the cathode during active flushing of the cathode compartment with water. (b) Schematic depiction of actively mitigating the buildup of ions and nucleation of crystal seeds on the catalyst by dissolving and removing salt from the cathode with water.

The second approach to reduce the consequences of salt precipitation works by actively adding solvents to the cathode region to dissolve and remove precipitates and elevated salt concentrations from near the cathode surface. While preventing salt formation is ideal, this second strategy demonstrates how operational performance can be regained after salts have precipitated in a CO_2_RR system. Importantly this strategy takes advantage of the fact that the most detrimental effect of salt formation is blockages of the CO_2_ diffusion pathways and not necessarily the nucleation of salt crystals themselves. If the salt crystals at the cathode can then be removed through the timely introduction of a secondary flow, the operational lifetime of the system can be increased (Figure 3). Additionally, preventative addition of water to the cathode region can periodically lower ion concentrations prior to salt formation occurring.

Endrődi et al. performed two experiments to remove the accumulation of K_2_CO_3_ salts in the cathode.^42^ In the first experiment, the cathode gas feed was humidified and heated to 85 °C to increase the water vapor in the cathode flow field and salt solubility. This approach allowed for stable operation for at least 8 h (at 200 mA cm^–2^, 3 V_cell_) but lowered the selective CO conversion to 65–70% due to the increased water content which promoted HER. In the second experiment, the cathode chamber was flushed once per hour with a 50 cm^3^ deionized water (*T*_cell_ = 60 °C). During CO_2_RR, a continuously decaying current (275–200 mA cm^–2^) was obtained, which the authors attributed to the formation of K_2_CO_3_. After each dissolution step, the reduced current returned to its initial value after which a new “decay cycle” was initiated. The combination of lower temperature and salt dissolution resulted in a continuous selectivity of 85% CO_2_-to-CO. The empirically chosen value of 50 cm^3^ deionized water shows that this method of regeneration is possible but also far from optimized. Later work by the same group cast doubt on cathode rinsing as a viable long-term technique for removing precipitates since significant pressure is necessary to penetrate the hydrophobic cathode and effectively clean out the precipitated salts.^20^ Currently, carbon-based GDEs commonly used for CO_2_RR are only mechanically robust enough to withstand pressure differences up to 100 mbar prior to flooding.^41,50^ Moreover, droplets that remain in the GDL after rinsing can promote HER and limit the free accessibility of CO_2_ to the catalyst. The two aforementioned effects indicate the limited feasibility of dissolution as a viable technique to overcome salt formation.

Instead of using water to periodically dissolve and flush out already formed salts, increasing the water availability has also been shown as a technique to prevent salt precipitation. In one case, De Mot et al. introduced more liquid water to a Sn-based MEA for formate production by injecting a constant stream of water with the cathode gas feed.^40^ The water injection rate was calculated by conducting a water balance on the cathode compartment, and the authors determined 0.15 mL/min of additional water was necessary to prevent salt precipitation. This calculation was in good agreement with their experimental results which found that at 0.2 mL/min of water injection, there was no visible salt formation within 1 h (although potassium was detected in the electrode pores by ICP-MS). For comparison, at a 0.1 mL/min water injection rate, the MEA failed after 50 min because of salt precipitation. Further increasing the water injection rate decreased the amount of K^+^ detected in the cathode GDE but also diluted the concentration of formate in the product stream. Typically, concentrated product streams are desired for downstream processing steps, so this work highlights the potential negative impact of water (and salt) management schemes on product dilution.

In a separate work, Wheeler et al. humidified the cathode gas feed to reduce the formation of salt precipitates.^12^ When water is supplied through the gas stream, less water is drawn across the anion exchange membrane to facilitate CO_2_RR. This means that co-ion transport of K^+^ across the membrane is reduced, mitigating the accumulation of K^+^ at the cathode. However, Mardle et al. noted that humidifying the gas feed lowers selectivity for CO_2_RR at higher current densities because of flooding of the cathode. Thus, water management is key to not only CO_2_RR performance but also salt precipitation.^51^ Conversely, others suggest that salt formation is initially caused by flooding of the electrolyte into the GDE and then drying of the electrolyte to leave behind salt crystals that subsequently pump more liquid into the GDE.^36^ So the question remains whether salt formation in the cathode GDE is caused by flooding and drying of the electrolyte, by salt crystals first forming and then pulling liquid in to flood the electrode, or a combination of both processes.^6,52^

The examples discussed above all use a liquid anolyte containing KOH or KHCO_3_ and rely on introducing more water to the cathode to flush out salts or limit co-ion migration. Recently, Endrődi et al. have successfully mitigated salt precipitation by taking the opposite approach: feeding the cell with a pure water anolyte and periodically “activating” the cathode by injecting a small volume of alkali cation containing solutions (10 cm^3^ of 0.5 M KOH) into the cathode feed.^20^ These solutions were 1:3 isopropanol/water mixtures (to help the solution penetrate the hydrophobic GDE) and were injected every 12 h of operation. At a constant cell potential of 3.2 V, initial introduction of the activation solution increased *j*_CO_ from 120 mA cm^–2^ to 350 mA cm^–2^. Over the course of 224 h, *j*_CO_ stabilized to 420 ± 50 mA cm^–2^ and no salt precipitation was observed in the cells; however, stable operation over thousands of hours using this technique has not yet been demonstrated.

## 3 Active Pulse Approach: Pulsed Electrolysis

**Figure 4:**
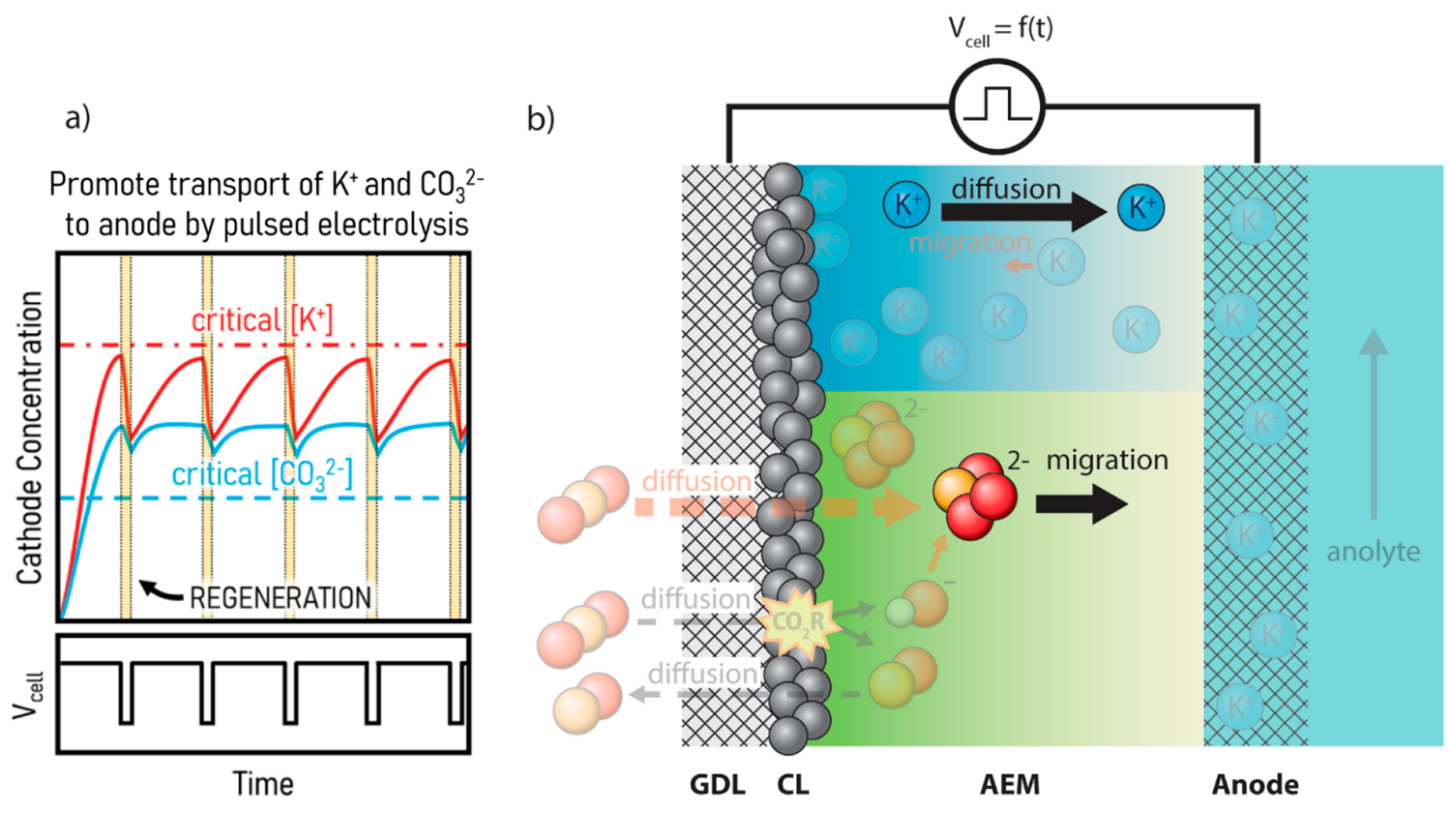
(a) Plots of cathode concentration and cell voltage versus time showing the general trends of K^+^ and CO_3_^2–^ concentrations at the cathode during pulsed electrolysis. (b) Schematic depiction of ion-transport during a “pulse” of lower voltage. At the lower regeneration voltage, the reaction slows down and migration of carbonates and K^+^ allow the system to partially homogenize before returning to the operational voltage.

A third approach to overcome salt precipitation is the use of a periodic regeneration voltage to redistribute ions within the MEA. In this approach the device voltage is ramped up and down in a predefined duty cycle, which lowers the operating current density and temporarily reduces the formation of byproduct hydroxide. During the lower voltage cycle the transport of ions in the system is maintained, however (Figure 4). Migration of K^+^ from the anolyte is then decreased, while CO_3_^2–^ has additional time to move to the anode, collectively decreasing the concentrations of both ions and preventing salt formation.

Xu et al. demonstrated the benefits of a recurring regeneration step where the potential was alternated between −3.8 V_cell_ during operation and −2.0 V_cell_ during regeneration. Stable operation was maintained for 236 h (out of which 157 h were at an operational voltage).^44^ When the same setup ran without a regeneration voltage, the system broke down after ∼10 h due to salt formation. Subsequent modeling of these two systems indicated that electromigration (instead of diffusion) of carbonate ions during the regeneration step is responsible for the long-term stability of the pulsed electrolyzer. These works indicate that active manipulation of applied current or voltage are viable methods of controlling the pH and ion distribution in an MEA to mitigate salt precipitation.

Due to the low number of case studies on altering operational and regenerative voltages as well as cycle durations, there is plenty of room for further investigation using this approach. To complement the relevance of this direction of research, future CO_2_ electrolyzers are likely required to operate intermittently to account for fluctuating power generation from renewable sources.^53^ However, there may then be too many operational constraints from both the electrolyzer and system perspective to optimize both fully.^54^

## 4 Passive Membrane Approach: Membranes and Materials

**Figure 5:**
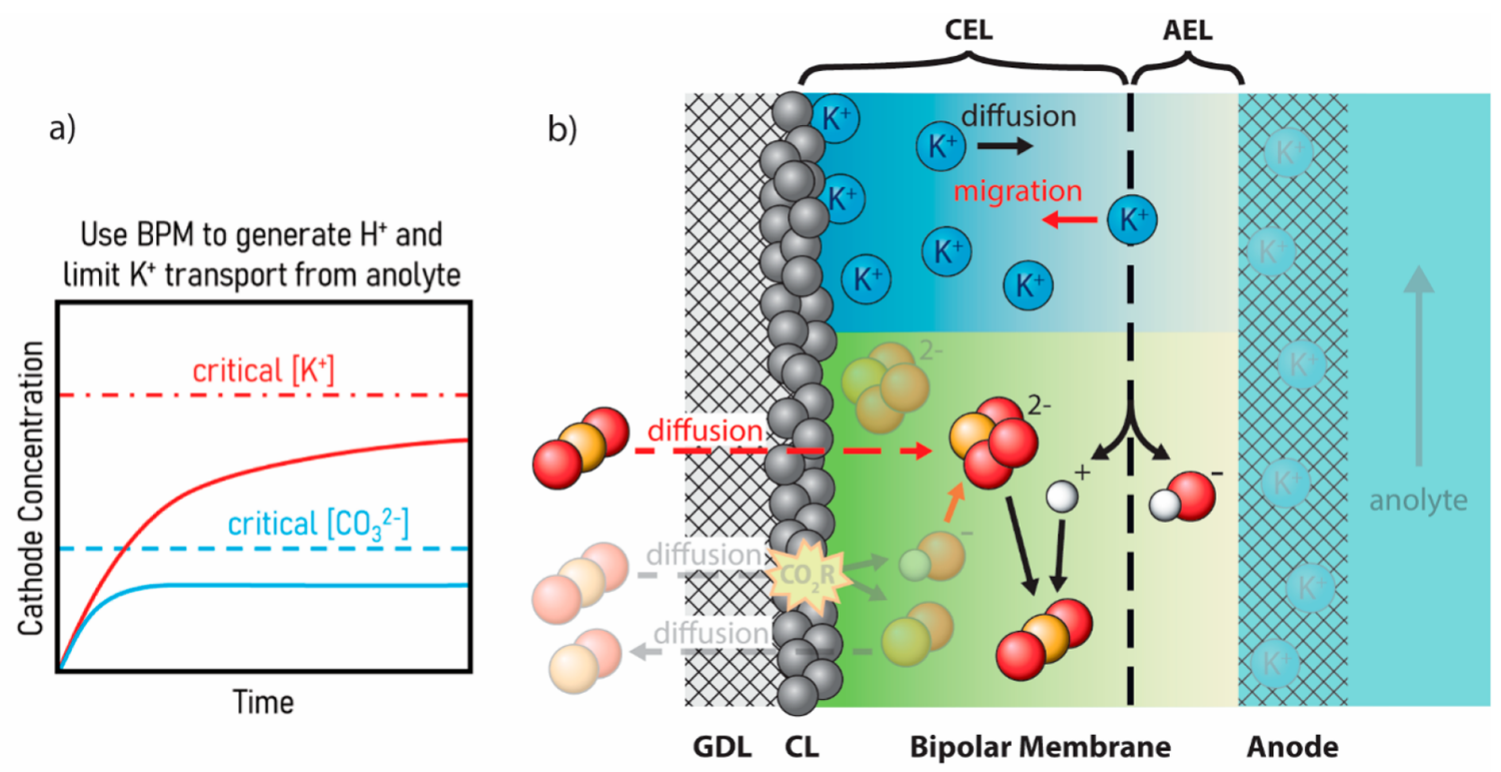
(a) Plot of cathode concentration versus time showing the general trends of K^+^ and CO_3_^2–^ concentrations at the cathode when a BPM is used. (b) Schematic showing effects of a BPM on K^+^-comigration past the membrane by limiting free ion transport and electro-osmotic drag. Additionally, CO_3_^2–^ concentrations are reduced by combining with H^+^ formed at the BPM junction to regenerate CO_2_. While changing the MEA recipe delays the accumulation of ions, it does not necessarily prevent critical concentrations from being reached.

The previous three approaches, while viable to maintain steady operation, all allowed for the excess formation of carbonate species. The operational approaches then provide an engineering solution rather than a fundamental solution to the problem of salt formation. The final approach described here aims to reconvert any formed (bi)carbonates back into CO_2_ by providing protons to the cathode chamber through the use of a bipolar membrane (BPM) instead of a monopolar membrane (Figure 5).^55−58^ Such an approach then adjusts the physical and chemical components of the MEA itself which differs from the previous operational approaches.

A BPM is composed of both a cation exchange layer (CEL) and an anion exchange layer (AEL) that are affixed to one another. Upon the application of a reversed bias (where the cation exchange layer is closest to the cathode and the anion exchange layer is closest to the anode), water inside the membrane is split into H^+^ and OH^–^ molecules which migrate to the cathode and anode, respectively. By using a BPM in a MEA for CO_2_ electrolysis, salt formation is then reduced through two different approaches. First, the H^+^ generated in the BPM migrates to the cathode and chemical interacts with (bi)carbonates to regenerate CO_2_, effectively offsetting the hydroxide that was generated in eq 1.^59−61^ And second, as H^+^ becomes the primary charge carrier, the migration of the co-ion K^+^ from the anolyte is greatly reduced. Both [K^+^] and [CO_3_^2–^] are then reduced using a BPM operating in reversed bias as compared to a monopolar membrane. A large factor in the success of using BPM’s to prevent salt formation resides in the ability to prevent co-ion crossover of potassium from the anolyte. Such BPM properties have been examined by Blommaert et al., who showed that under reversed-bias conditions water dissociation will dominate K^+^ co-ion crossover at current densities >10 mA cm^–2^.^62^ In fact, beyond current densities of 1 mA cm^–2^, the flux of K^+^ was shown to be fixed almost independent of the applied current density and constituted less than 3% of the charge transported across the membrane. Thus, a BPM is likely to greatly slow salt precipitation by limiting potassium transport to the cathode but by itself will not clearly avoid precipitation.

In literature, the reversed-bias BPM approach has been used in a number of scenarios with the primary intent to increase CO_2_ utilization within CO_2_ electrolysis systems.^21,56,63−65^ If a higher fraction of CO_2_ is used for the electrochemical reaction, then less CO_2_ can be permanently converted into carbonate salts. Interestingly, the BPM configuration does not avoid carbonate formation which could lead to salt formation but instead provides a means of neutralizing the formed carbonate with protons prior to the anions migrating through the cation and anion exchange layers. The BPM approach has then allowed for stable operation of >12^55^ and 24^56^ hours in two examples, with several others reporting much more stable operation than with anion or cation exchange membranes alone.^21,64,65^ The use of BPM’s in reversed bias, however, has an associated energy cost. Specifically, BPMs require increased potentials to dissociate water at the anion and cation exchange membrane interface. Additionally, the presence of two membranes causes greater charge ohmic resistance than a singular thinner membrane. Further designs of effective bipolar membranes might help in overcoming the higher cell voltages encountered in commercially available BPMs.^65^ Promising results by Oener et al. for example showed that optimization of the BPM through lower thickness, increased AEM/CEM interface area and an additional water dissociation catalyst inserted at the cation and anion junction led to overpotentials as low as 10 mV (at 20 mA/cm^2^).^66^ As further work continues on BPMs, their potential to reach elevated current densities at lower overpotentials is expected then to increase.

A secondary issue with using BPMs in reversed-bias is that the cathode conditions become acidic. Without proper control of the cathode pH and mobile cations, hydrogen evolution can then outcompete CO_2_ reduction. Some approaches have used a weakly acidic buffer layer to increase the pH to a point where CO_2_ electrolysis is favorable again.^56,65,67^ Such results motivate further reinvestigation into acidic CO_2_ electrolysis catalysts.

While less common, systems for CO_2_ conversion have also considered using BPMs in a forward-bias configuration. When operating a BPM in forward bias (where the AEM is pressed against the cathode compartment instead) some energy can be recovered by the recombination of ions at the cation and anion exchange junction. Here CO_2_ can be regenerated as carbonate from the cathode and protons from an acidic anolyte recombine. Such an approach, while preventing salt precipitation through the use of an acidic anolyte, causes gas evolution in the middle of the membrane.^68^ In principle, a monopolar CEM can also be used to transport H^+^ ions toward the cathode using an acidic anolyte solution devoid of cations. The acidity of the cathode needs to be balanced, however, to avoid excessive proton concentrations which would cause HER to dominate CO_2_ electrolysis.^69^ Additionally cations are likely necessary for CO_2_ electrolysis to outcompete hydrogen evolution.

A common challenge for CO_2_RR research is controlling the environment close to the catalyst such that the core performance metrics of voltage, current density, selectivity, and stability can all be maintained. The issue of salt precipitation in MEA systems is no exception and requires consideration of the electrochemical and chemical reactions occurring in the system, as well as mass transport within each component. Herein we identified several mechanisms that lead to salt formation and reviewed four operational techniques for salt precipitation prevention in neutral and alkaline CO_2_RR MEAs, all with the goal of lowering cation and/or carbonate concentrations near the cathode.

The outlook for each of the presented approaches are promising given the relatively few papers that have tried to directly address salt formation, leaving room for greater advancements. For example, there remains a large amount of operating conditions left to be tested, and combining a subset of the approaches above is likely to allow for salt formation failure to be prevented indefinitely. It is also worth noting that the challenges associated with salt and carbonate formation were only noted a few years prior to this article, and there are now several proposed solutions, highlighting progress in a short period of time. Notably for each of the presented cases, however, is that system stability was improved at the cost of decreases in other performance metrics. For example, decreasing the anolyte concentration or using a BPM is penalized by higher cell voltages, while periodic operation lowers the capacity factor of the electrolyzer. Future work then needs to evaluate which trade-offs are acceptable at the expense of other metrics.

Looking to the future, we note that operational strategies are not the only methods available to stop salt precipitation, and we expect materials selection and development to also play a role. Recent reports in flow cells have demonstrated the ability of ionomer binders, monolayers, and bilayers to control local concentrations of ions in the catalyst layer and influence salt precipitation.^5,70^ When developing solutions to overcome salt precipitation for CO_2_RR, researchers can also look to other fields for inspiration. Research on durable membranes for water filtration applications has extensively studied material design strategies (i.e., controlling surface charge, roughness, hydrophobicity, etc.) to mitigate membrane fouling by inorganic salts (primarily CaCO_3_, SiO_2_, and BaSO_4_).^71^ The formation of carbonate salts at gas–liquid–solid boundaries is also of interest to geological carbon storage applications whereby CO_2_ is injected into saline aquifers for sequestration.^72^ Further study into salt nucleation and growth mechanisms under CO_2_RR conditions by *operando* or *in situ* characterization techniques (i.e., atomic force microscopy, nano- or microcomputed tomography, X-ray diffraction, etc.) will also inform the development of both materials-based and operational salt prevention strategies. Lastly, the emerging field of CO_2_RR under acidic conditions provides another avenue to avoid the issue of salt precipitation entirely, as the reduction of hydroxide concentrations leads to significantly less homogeneous formation of salts.^73^ Future works should weigh the advantages and disadvantages of CO_2_RR in acidic and alkaline environments to determine which system is most desirable for a durable and selective electrolyzer operating at industrially relevant current densities, that also maintain overall high energy and carbon efficiency.

Salt precipitation is one of the major limitations for the selective and long-term operation of neutral and alkaline MEA CO_2_RR electrolyzers. This issue is difficult to avoid because the three essential components for CO_2_RR (CO_2_ gas, a proton source, and an alkali cation) also directly influence the local concentrations of ions that can precipitate into salt deposits. Here mechanisms for salt formation are discussed, and four operational approaches to prevent or reverse salt precipitation are presented, which can be broken down into either passive system changes or active mediation. Several of these strategies are successful over the course of tens to hundreds of hours; however, none demonstrate selective system operation on the order of tens of thousands of hours. We encourage researchers to report longer term electrolysis studies using these salt precipitation prevention methods and analyze their feasibility for commercial systems. A combination of operational solutions will likely need to be deployed to solve the salt precipitation problem.
